# Abstracts and Selections

**Published:** 1916-05

**Authors:** 


### Abstracts and Selections.

### Safeguarding Medical Ethics.

Under the head of "Contemporary Notes," the *New York Medical Journal,* which is one of the strongest and best journals published in the United States, quotes the following from this JOURNAL:

"Medical ethics is a good thing, according to the TEXAS MEDICAL JOURNAL for February, 1916, and the ethical ideals should be high. There is always danger, however, that harm may be done in the name of medical ethics. Let a member of a profession but suggest that a thing is unethical, and often it is condemned without a proper consideration of whether it is in fact unethical. Medical ethics should never be allowed to stand as a bar to progress in the science of medicine; neither should it be used as an excuse for one physician giving another an undeserved kick. Ethics, because it is such a popular term, is always in need of safeguarding from abuse. It is safe in the hands of those who always behave as gentlemen should behave, but the interpretation is so pliable that it is at times used as a weapon for wrongdoing."

### An Efficient Secretary of the Navy.

In his address before the graduating class of the Naval Medical School on April 12, 1916, the Surgeon General of the Navy, Rear Admiral William C. Braisted, reviewed the work of the present administration of the Navy Department in so far as it affected the Medical Corps of the Navy. The Surgeon General said:

"During the past two years, the present Secretary of the Navy, in many instances on his own suggestion and in others by his support of the Bureau of Medicine and Surgery, has advanced our service in the following items:

"1. He has recommended to Congress the increase of the Medical Corps from 347 to nearly 500, the first increase in twenty years and most urgently needed.

"2. He has provided in his personnel bill a substantial increase in the upper grades of the Medical Corps. No increase in these grades has been made since 1870, nothwithstanding the tremendous growth of the service as a whole.

"3. He has established two of the finest Hospital Corps Training Schools in the world for the training of male nurses, and made provision for the increase of this corps by nearly 11,000 men, an increase urgently demanded by the increase in the navy, and has also provided a possible chance for members of this corps to reach commissioned rank, thereby raising the efficiency and esprit of nearly 2000 enlisted men in the Hospital Corps of the Navy.

"4. He has made provision for a new hospital ship for the navy, which will enable us to build the first ship of its kind, designed especially for the purpose, and which will be a model for our country and the world. This one effort will, if granted by Congress, be of inestimable value to the navy and to all other countries. It will provide our fleet and our service with a floating hospital for the care of the sick and injured of the navy that will rival any metropolitan hospital in existence.

"5. He has established schools for the training of the native women of Samoa and Guam in nursing that already are giving most excellent results and are a most important humanitarian educational effort for these helpless people, promising, when completed, to be one of the noblest efforts made for the uplifting of any similar class of people.

"6. He has increased our appropriations to meet our necessities and to enable us to carry on our great work that extends not alone to the navy proper, but to thousands of human beings attached to the service in various ways and extending to all parts of the world.

"7. He has permitted us to take an active part in the regeneration of Haiti by furnishing medical officers and nurses to care for the sanitary needs of the great work that is now in progress in Haiti and which ranks with one of our greatest humanitarian efforts.

"8. He has supplied already, and gradually will furnish our deficiencies in the many large hospital and medical organizations on shore, such as contagious units at Mare Island, Puget Sound, New York and Newport, and has furnished adequate and commodious homes for our nurses at Mare Island and Boston, thus adding to the content and efficiency of the women nurses. South of Norfolk and San Francisco our coast line is practically unprovided with hospitals for peace and war, but with a policy defining permanent naval stations his attention has already been turned to the needs of the Medical Department. That we may not be unprovided in this respect in emergency, he has authorized our efforts with the Red Cross, and we are now beginning the organization of five Red Cross Hospital units (mobile hospitals of 250 beds each, with personnel and equipment complete) that can be called at notice to any point of this coast line where needed.

"9. He has authorized a Medical Reserve Corps, of the best medical talent that our country can furnish, to be ready to come to our assistance in time of need, and to prepare this group of the Naval Medical Corps is initiating a correspondence school that shall give these officers a training and working knowledge of their work when called upon. Realizing the great lesson to be learned from the present European war, he has detailed a number of our best officers for duty in Europe, as observers, and the excellent results already show in the report of Surgeon Fauntleroy, whose work has added greatly to the professional knowledge of all who are interested in medico-military matters and is a credit to our service.

"10. The above are only a few examples showing the interest and activity of our Secretary in this branch of the service. Did time permit, I should like to continue in detail to show you his interest and help as shown in innumerable questions of sanitation and organization which makes the work of the Medical Department of the Navy one of the most important branches of the service. Our work is most highly specialized, dealing as it does with so many questions, not alone with the healing of diseases, but with all that pertains to the orderly and successful running of this great department of the government. The work of the Bureau of Medicine and Surgery touches intimately that of every other bureau in the department and makes us one of the busiest and most active corps.

"I have frequently asked myself how is it that the head of the navy is so sanely interested in our work, and seems to comprehend our needs often before they are presented to him, and I think the solution lies in the fact of his education along medical lines. This comes, I feel sure, from the association with medical men of great worth and ability. Where has he had this association which has taught him the importance of medical work in the welfare of the nation? From all I can learn, it has come from the intimate friendship and association of his family medical advisers."

